# Simultaneous Sampling of Flow and Odorants by Crustaceans can Aid Searches within a Turbulent Plume

**DOI:** 10.3390/s131216591

**Published:** 2013-12-03

**Authors:** Swapnil Pravin, Matthew A. Reidenbach

**Affiliations:** 1 Department of Mechanical and Aerospace Engineering, University of Virginia, Charlottesville, VA 22904, USA; E-Mail: sp8yh@virginia.edu; 2 Department of Environmental Sciences, University of Virginia, Charlottesville, VA 22904, USA

**Keywords:** plume, olfaction, turbulence, tracking, crustacean, odorants

## Abstract

Crustaceans such as crabs, lobsters and crayfish use dispersing odorant molecules to determine the location of predators, prey, potential mates and habitat. Odorant molecules diffuse in turbulent flows and are sensed by the olfactory organs of these animals, often using a flicking motion of their antennules. These antennules contain both chemosensory and mechanosensory sensilla, which enable them to detect both flow and odorants during a flick. To determine how simultaneous flow and odorant sampling can aid in search behavior, a 3-dimensional numerical model for the near-bed flow environment was created. A stream of odorant concentration was released into the flow creating a turbulent plume, and both temporally and spatially fluctuating velocity and odorant concentration were quantified. The plume characteristics show close resemblance to experimental measurements within a large laboratory flume. Results show that mean odorant concentration and it's intermittency, computed as *dc/dt*, increase towards the plume source, but the temporal and spatial rate of this increase is slow and suggests that long measurement times would be necessary to be useful for chemosensory guidance. Odorant fluxes measured transverse to the mean flow direction, quantified as the product of the instantaneous fluctuation in concentration and velocity, *v′c′*, do show statistically distinct magnitude and directional information on either side of a plume centerline over integration times of <0.5 s. Aquatic animals typically have neural responses to odorant and velocity fields at rates between 50 and 500 ms, suggesting this simultaneous sampling of both flow and concentration in a turbulent plume can aid in source tracking on timescales relevant to aquatic animals.

## 1. Introduction

### 1.1. Chemosensing in Aquatic Animals

Crustaceans such as crabs, lobsters and crayfish use chemo- and mechano-reception to track sources of odorant plumes to locate mates, food, and living habitat [1–6]. Odorants in the benthic flow are carried to the olfactory organs of the animal through turbulent water currents and diffuse toward the surface of the organs where chemoreceptors are located. These olfactory organs also contain mechano-receptors that provide information about the turbulent flow, and together with odorant concentration help the animal locate the source of the chemical plume [7]. Animals use a variety of sensing strategies to orient themselves in the direction of the plume source depending on the flow regimes they operate in [8]. Hence, to understand the mechanism of chemical plume tracking in aquatic animals, we must understand not only the small scale diffusive flow of odorants near the olfactory organs of the animals, but also the large scale turbulent nature of the chemical plume.

**Figure 1.**
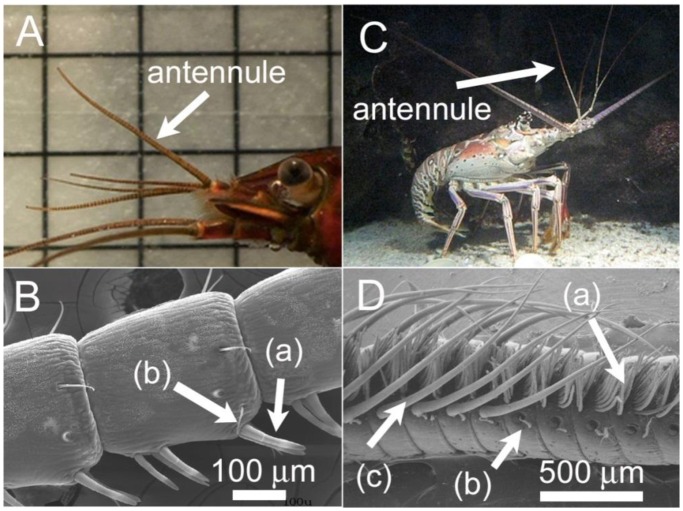
(**A**) The freshwater crayfish, *Procambarus clarkii*, with lateral antennule labeled. Grid in the background is 1 × 1 cm; (**B**) Scanning electron micrograph (SEM) of the lateral antennule with (a) chemosensory aesthetascs and (b) mechanosensory sensilla labeled (photo D. Mellon); (**C**) The spiny lobster, *Panulirus argus*, with the lateral antennule labeled; (**D**) SEM of the lateral antennule with (a) chemosensory aesthetascs; (b) mechanosensory sensilla and (c) guard hairs labeled (photo J.A. Goldman).

Crustaceans have olfactory appendages called antennules, which bear tiny hair-like structures called aesthetascs (Figure 1). The aesthetascs are often covered by a permeable cuticle membrane underneath which reside chemoreceptors. The chemoreceptors contained on the aesthetascs are composed of dendrites (branched projections) of olfactory receptor neurons (ORNs), which send information, through electrical impulses, to the olfactory lobes of the brain [9]. The diffusion of odorant molecules toward and through the cuticle membrane is responsible for delivering the odorants to these chemoreceptors. To facilitate the transport of odorant laden flow to the antennules, many aquatic animals use flicking or fanning of their appendages [5,10]. This behavior is often described as “sniffing”. A flicking motion often involves a fast down-stroke and a slower return-stroke, leading to entrapment of odorant molecules between the aesthetascs, which lowers the diffusion time of odorants to the aesthetasc surface and enables these animals to discretely sample their ambient environment [11,12].

The odorant plumes encountered in the environment of these organisms are often turbulent and highly filamentous in nature [6,13]. Due to stirring by the turbulent motion of the fluid, the spatial and temporal distribution of odors is complex and filaments of high odor concentration are often adjacent to little or no odorants [13,14]. These distributions in odorants also change in response to variations in the ambient flow speed and bed roughness, where the variance in odorant fluctuations is reduced for rougher beds [15] and greater mean velocities [13]. Certain cues, such as correlations between the flow kinematics and odorant concentration that the animal sense through the chemoreceptors and mechanoreceptors, can provide valuable information regarding the plume source. However, due to the high intermittency and temporal and spatial variability of the plume, this often reduces the ability of organisms, such as the blue crab, *Callinectes sapidus*, to successfully navigate to the source of an attractive odor [16].

### 1.2. Sampling Rates and Tracking Strategies in Crustaceans

The frequency of flicking and sensitivity to odorants can alter the tracking strategy in the animals. Higher sampling frequency allows an animal to sample a larger number of odorant filaments as it moves through the plume [13]. Blue crabs *C. sapidus*, spiny lobsters *Panulirus argus* and freshwater crayfish *Procambarus clarkii* all flick their antennules at a rate of approximately 3 Hz, but can vary between 0.5 and 4 Hz [13,17]. Although it was previously assumed that most odor tracking by animals occurred by responding to time-averaged concentration gradients in a plume [18], the speed at which plume-tracking maneuvers occur suggest that more instantaneous sensory feedbacks are being utilized [8,19], and that time-averaged concentrations converge too slowly to be useful to a foraging animal [20]. In a similar fashion, it was also found that resolving the rise slope of concentration bursts requires sampling rates that are too fast for animals and the spatial variation in the rise slope is too small to be utilized in search without long sampling periods. Overall, the time-averaged concentration, rise slope, and burst shape of concentration filaments, if utilized alone, have limited usefulness for plume tracking [20].

Page *et al.* [21] found that in blue crabs, odorants elicit responses in a binary way, causing upstream motion provided that the concentration detected along the antennules exceeds a specific threshold. However, this threshold was different for each crab as well as different due to the prior stimulus history, suggesting a context-sensitive response to signal dynamics. In addition, Page *et al.* [22] found that the spatial distribution of the odorant concentration field was utilized to correct for cross-stream motion, and that crab movement is continually adjusted to maintain an upstream heading in response to both the concentration and its distribution. While many organisms using this flicking of their antennules to discretely sample the plume at short timescales [23], some slower moving predators such as the knobbed whelk *Busycon carica* have been found to use temporal averaging to gather information on the spatial extent of the plume [24]. Whether this temporal averaging is used widely by aquatic organisms, or how useful it is within highly turbulent plumes, is still unknown.

### 1.3. Neural Responses to Odorant and Flow Information

Previous studies have shown that the time course of advection and molecular diffusion of odorants to chemoreceptors can play a significant role in neural responses [25,26]. For example, the olfactory receptor neurons in the lobster *Homarus americanus* require at least 50 ms of odorant stimulus to fire, but 200 ms or more for the spike frequency to fully respond to the concentration of the odor pulse [27]. However, lobster antennule receptor neurons start to adapt after continuous exposure to an odor stimulus of 300 ms and are completely adapted after 1,000 ms of exposure. This adaptation resets the sensitivity of neuron response to odorant concentration higher than background levels. Neuron responses in the spiny lobster *Panulirus argus* show decreased sensitivity when continually exposed to odorants at repeated odor pulses between 100 ms to 500 ms [28]. This suggests that both intermittency in the odorant signal within the plume and discrete sampling of odorants by the animal are highly beneficial for detection of odorants and affect the rate of receptor neuron firing [27,28].

Hydrodynamic stimulation of the antennules, including flicking [29,30] and from ambient current evokes electrical activity in the central brain neurons [31–33]. However, peak responses of olfactory receptor neurons occur not solely when the aesthetasc responds to odorants, but rather in conjunction with a hydrodynamic stimulus [31,34,35]. Thus, concentration and flow cues simultaneously excite chemoreceptors and mechanoreceptors of the antennules during flicking. In addition, many invertebrate organisms, including the spiny lobster *P. argus* [36], and crayfish *P. clarkii* [7], contain bimodal chemo-mechanoreceptors on their antennules that respond to both odorants and flow. For *P. clarkii*, local deutocerebral interneurons integrate hydrodynamic and odorant inputs, and the response of these central elements is enhanced when both flow and odorant stimulation occurs together [31]. For the crayfish *Orconectes virilis* changes in the temporal pattern of odorant stimulation was found to drive changes in the temporal patterns of behavior while undergoing search [37]. It has also been shown in blue crabs *Callinectes sapidus* that both rheotaxis and chemotaxis is necessary for successful orientation while tracking food odors [38]. These findings suggest that these organisms utilize both hydrodynamic and chemical stimuli to aid in search behavior, but what chemical and flow information these organisms use, and how it is integrated at the level of the antennule or brain remains poorly understood.

### 1.4. Research Objectives

The objective of this study is to determine the impact of ambient flow environments on turbulent mixing and odorant transport within a chemical plume, and how crustaceans might use simultaneous flow and odorant information to orient themselves within a plume. The specific questions that are addressed in this study are: (1) How does bed roughness and water velocity impact turbulence structure and mixing of dissolved odorants? (2) What is the effect of flow kinematics on mean and instantaneous flux of odorants at different downstream and transverse locations within a turbulent plume? (3) Since antennules of aquatic crustaceans contain both chemo- and mechano-sensory sensilla, can they conceivably integrate velocity and concentration information to aid in finding the location of the plume source? We utilize simultaneous measurements of water velocities and odorant concentrations over a sand bed roughness within a large laboratory flume, as well as numerically simulate a similar turbulent plume using a 3-dimensional computational fluid dynamics model to address these questions. Understanding the potential mechanisms by which these organisms locate sources of odor can also provide inspiration for artificial bio-mimetic sensors.

## 2. Methods

### 2.1. Flume Study

**Figure 2.**
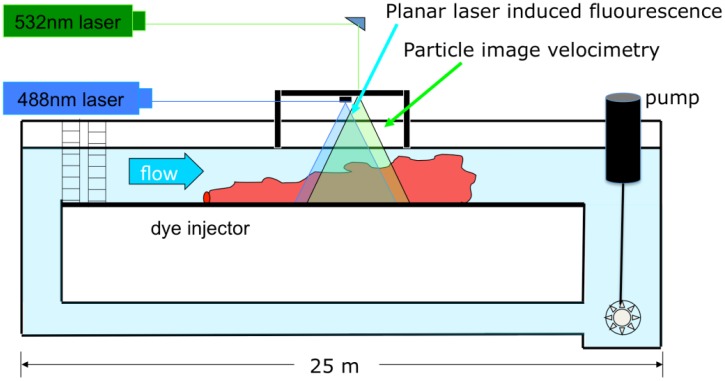
Recirculating flume with PLIF/PIV laser system. A 532 nm laser was used for particle image velocimetry, while a 488 nm laser was used to excite fluorescein dye for use in PLIF imaging. Images were obtained 13 m downstream from the leading edge of the flume and 1 m downstream from the source release of fluorescein dye.

The experimental data for flow and odorant structure within a turbulent plume were analyzed from a study conducted within a rectangular laboratory flume (Figure 2) with dimensions 25 m long, 0.6 m wide, and 0.3 m high [39]. Velocity measurements were made using a Particle Image Velocimetry (PIV) system while concentrations were sequentially obtained using a coordinated planar laser-induced fluorescence system (PLIF). The PLIF technique, described in further detail in [13], uses fluorescein dye as the scalar tracer. A laser with an output wavelength of light at 488 nm was first passed through a beam expander, then a beam focus in order to sharpen the beam. The beam was then converted to a vertical sheet of laser light using a scanning moving-magnet mirror (Cambridge Technology Inc., Bedford, MA, USA). The frequency of laser light was within the absorption spectrum of fluorescein (mean excitation at 490 nm), which excited the dye and emitted light at a mean wavelength of 520 nm. Using a digital camera (1M60, Dalsa, Waterloo, Canada), 1 megapixel, 12 bit resolution, 50 frames s^−1^), the fluoresced dye was imaged. The camera was fitted with an optical longpass filter which passed all light above 517 nm (Omega Optical Corp., Brattleboro, VT, USA), and therefore only emitted light from the fluoresced dye was imaged, while the ambient laser light was blocked. Imaging occurred over an approximate 8 cm × 8 cm area 1 m downstream from the point of odorant release along the centerline of the plume. The laser was scanned to illuminate the imaging field every 0.04 s (25 Hz), with a wait period of 0.04 s between scans. Raw images were processed to remove biases in the data, including varying pixel dark response, slow background changes in pH and temperature, lens and optics aberrations, and laser attenuation due to background concentrations [40].

A second laser with an output wavelength of light at 532 nm was used for PIV imaging. PIV is a standard technique [41] in fluid flow applications in which a thin sheet of laser light illuminates a two dimensional plane within a flow. Silver coated hollow glass spheres (diameter = 11 μm) were added to both the dye and bulk flow and the 532 nm laser was used to illuminate the particles. Since the 532 nm laser light was outside the absorption spectrum of fluorescein dye, the dye did not fluoresce. The laser was also pulsed at 0.04 s intervals, alternating in time with the PLIF laser scanning. Particle motions illuminated by the PIV laser were recorded with the same camera as the PLIF images in alternating frames [42]. Images of particle trajectories were analyzed using a numerical method that employs cross-correlation analysis to calculate the most likely displacement of particles over a given time period between image frames [43]. The MatPiv 1.6.1 software package [44] was used to calculate velocity vectors for every 8 by 8 pixel subwindow, giving a velocity resolution to 0.6 mm, while concentration information was on the pixel scale, with a resolution of <0.1 mm. Vector fields were superimposed on the sequentially-recorded scalar concentration field.

A dye source concentration *C_source_* = 100 μg cm**^−^**^3^ was used as the tracer. Fluorescein has a Schmidt number, *Sc* = 1,970 at 20 °C. Most odors which attract aquatic organisms are composed of amino acids with a molecular diffusivity, *D* ≈ 10^−9^ m^2^ s^−1^, making fluorescein, with a molecular diffusivity of *D* = 0.5 × 10^−9^ m^2^ s^−1^ a good choice as a surrogate to model odor transport. Dye was injected uniformly across the width of the flume along the sediment-water interface using a line source diffuser. The diffuser, constructed of 1 cm diameter Tygon^®^ tubing, released dye from 2 mm diameter ports spaced evenly in 2 cm increments across the width of the plume. Fluorescein input was from a constant head tank located above the flume at a rate of 0.04 L min^−1^. This type of release created a uniform distribution of dye across the width of the flume while minimizing flow disturbances and mixing effects due to introduction of the dye. Measurements were made at 1 m downstream from the source. One flow condition, of approximately *U* = 10 cm s^−1^ was quantified over a bed composed of sand with a mean grain size diameter of 1.5 mm. Due to memory capacity of the camera, 1,000 sequential images were collected in each run, equivalent to ∼20 s at a 50 Hz sampling rate. 10 runs were completed for each flow condition, therefore 10,000 total images were obtained, containing 5,000 PIV images and 5,000 PLIF images.

### 2.2. Numerical Model

**Figure 3.**
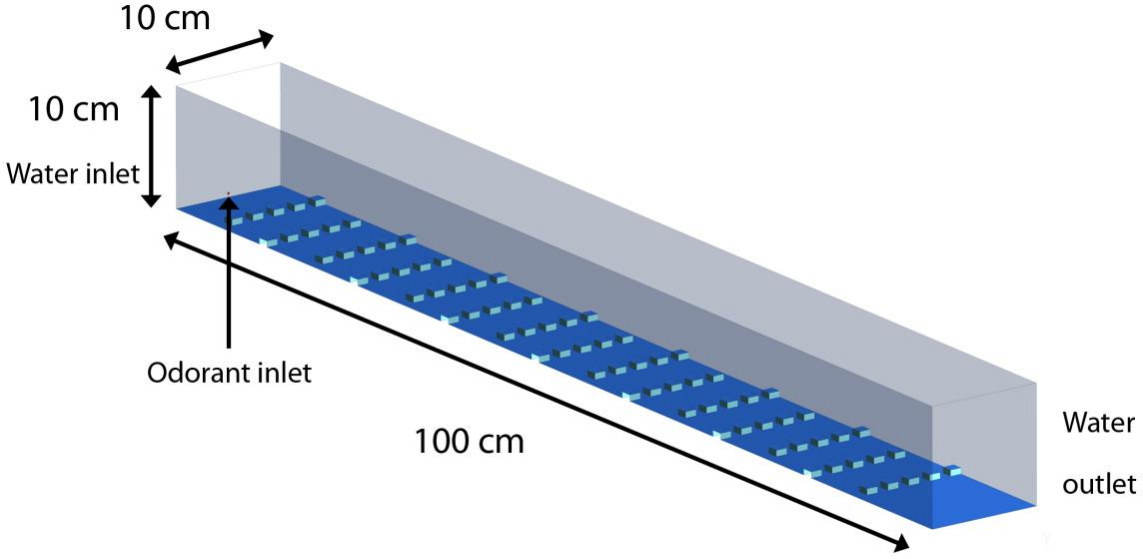
Schematics of the CFD domain for flow simulation. The bottom surface of the three-dimensional domain has cubical roughness elements placed in a staggered fashion. Water enters from the left end and exits from the right. The roughness humps at the bottom, of side length 1cm and separated by 5 cm from each other, help trip the boundary layer and facilitate mixing leading to a turbulent flow downstream. A stream of odorants is released into the inflow at the odorant inlet on the left at an elevation of *z* = 0.5 cm. The cubical humps have a base area of 1 cm by 1 cm and a height of 0.5 cm to hydraulically reproduce a sand bed roughness.

To simulate the turbulent plume measured within the laboratory flume, a 3-dimensional numerical model was developed that uses cubical roughness elements placed at the bottom surface of the flume to generate turbulence characteristics within the water column similar to the sand bed topography of the flume experiments. The cubical roughness elements were 0.5 cm high and the distance between successive rows of roughness elements was 5 cm. Each successive row was placed in a staggered arrangement with respect to the previous row allowing continuous water movement in the region between the roughness elements. This creates a roughness height (*h*) to spacing (*d*) ratio of *d*/*h* = 10, forming a *k*-type roughness, similar to that created by the sand bed [45]. Figure 3 shows the flow domain where water enters the left end of the flume and exits from the right end. A plume of odorants was injected into the flow domain from the left boundary, 3 mm above the bed, through a 2 mm wide opening. The grid size for the mesh varied throughout the domain to allow for a fine resolution of boundary layer flow near the roughness elements. The smallest grid size was 0.05 cm near the surface of the roughness elements. A large-eddy simulation (LES) technique was used for modeling turbulence in the flow, which allowed for accurate resolution of large eddies, while the Smagorinsky eddy-viscosity model was used for modeling small scale eddies. The governing equation for the flow is given by the Navier–Stokes equation:

(1) $$\frac{\partial u_{i}}{\partial t}+u_{j}\frac{\partial u_{i}}{\partial x_{j}}=-\frac{1}{\rho }\frac{\partial p}{\partial x_{i}}+\upsilon \frac{\partial^{2}u_{i}}{\partial x_{j}\partial x_{j}}$$

where *ρ* is the density and *υ* is the dynamic viscosity of water. The continuity equation for incompressible flow, along with the equation above, defined the velocity field:

(2) $$\frac{\partial u_{i}}{\partial x_{j}}=0.$$

Using velocity data, the odorant concentration, *c*, was calculated at each time-step using the mass transport equation:

(3) $$\frac{\partial c}{\partial t}+u_{j}\frac{\partial c}{\partial x_{j}}=D\frac{\partial^{2}c}{\partial x_{j}\partial x_{j}}$$

where *D* = 10^−9^ m^2^ s^−1^ [46], equal to the diffusivity coefficient of amino-acid derived odorants in water. The full Navier–Stokes equation was solved for velocity, pressure and odorant concentration using the CFX™ solver on a mesh of 3.3 × 10^6^ nodes. For eddies smaller in size than the grid size, a filter was applied using: *u*=*u̅*+*u′*, where *u̅* is the filtered velocity and *u′* is the residual velocity. The governing equation for the filtered velocity *u* and scalar variable *c* are given by:

(4) $$\frac{\partial {\bar{u}}_{i}}{\partial t}+\frac{\partial }{\partial x_{j}}\left({\bar{u}}_{i}{\bar{u}}_{j}\right)=-\frac{1}{\rho }\frac{\partial \bar{p}}{\partial x_{i}}+2\frac{\partial }{\partial x_{j}}[\left(\upsilon +\upsilon_{R}\right){\bar{S}}_{ij}]$$

(5) $$\frac{\partial \bar{c}}{\partial t}+\frac{\partial }{\partial x_{j}}({\bar{u}}_{j}c)=\frac{\partial }{\partial x_{j}}\left[(D+D_{R})\frac{\partial c}{\partial x_{j}}\right]$$

The eddy viscosity is modeled as $\upsilon_{T}={\left(C_{s}\Delta_{g}\right)}^{2}\sqrt{2{\bar{S}}_{ij}{\bar{S}}_{ij}}$, where ${\bar{S}}_{ij}=\frac{1}{2}\left(\frac{\partial {\bar{u}}_{i}}{\partial x_{j}}+\frac{\partial {\bar{u}}_{j}}{\partial x_{i}}\right)$ is the strain-rate tensor and Δ*_g_* is the grid size. The eddy diffusivity is *D_R_*= *υ_R_*/*Sc*. Two velocities were studied, with a uniform inlet speed of *U_0_* = 10 and 15 cm s^−1^, which corresponds to Kolmogorov scales of η = 1.3 and 1.0 mm and Batchelor scales of λ_B_ = 0.03 and 0.02 mm respectively. The boundary conditions for the model were:

*Inlet*: water entered at a uniform speed of *U_0_* (= 10 and 15 cm s^−1^). The odorant stream entered the flow domain 3 mm above the bed through a circular opening (2 mm diameter) at a speed of *U_0_* and a concentration of *c_source_*= 100 μg cm^−3^.
*Outlet*: water and odorants exited the flow domain at a fixed outlet pressure of *p* = 1 atm.
*Sides and top*: the normal components of the velocity and scalar variable gradients were set to zero: *∂u*/*∂n*=0, *∂c*/*∂n*=0.
*Bottom*: no-slip wall boundary condition was applied at the bottom boundary: *u_x_* = *u_y_* = *u_z_* = 0.

## 3. Results

### 3.1. Laboratory Flume Studies

**Figure 4.**
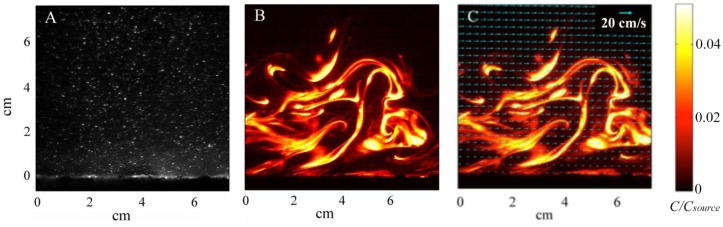
(**A**) Image of particle-laden fluid used in the PIV analysis; (**B**) PLIF image of plume structure 1 m downstream of the source; (**C**) combined plume structure from PLIF and velocity vectors (shown as blue arrows) calculated from PIV cross-correlation analysis. Odorant was released 1 m upstream from the imaging area by emitting fluorescein dye from a 1 cm tygon tube embedded across the width of the flume in the sandy bed material. Flow is from left to right.

**Figure 5.**
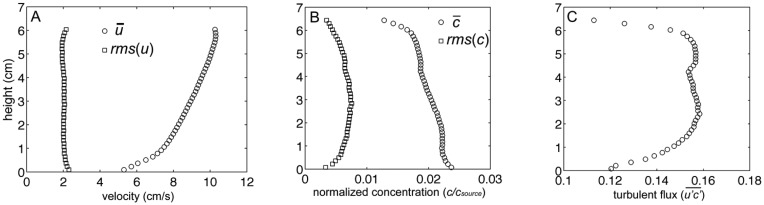
(**A**) Mean and fluctuating components of horizontal velocity within the laboratory flume from PIV. Fluctuating component computed as the root mean square (*rms*) of the horizontal velocity; (**B**) Mean and *rms* fluctuating component of normalized concentration from PLIF; (**C**) Mean horizontal turbulent flux (g cm^−2^ s^−1^), computed as mean of the fluctuating horizontal velocity, *u′*, (in cm s^−1^), multiplied by the fluctuating normalized concentration, *c′*.

Instantaneous particle motions from PIV and dye concentrations from PLIF are shown in Figure 4A,B respectively. Velocity vectors, computed from PIV analysis, were superimposed over the PLIF image to quantify simultaneous water velocities and odorant concentrations 1 m downstream from the source, along the plume centerline (Figure 4C). PLIF images show the highly filamentous structure and inherent intermittency in the turbulent odorant signal. From these PIV and PLIF images, instantaneous velocities and concentrations were separated into time-averaged and turbulent fluctuations of horizontal velocity, *u*=*u̅*+*u′*, vertical velocity, *w*=*w̅*+*w′*, and concentration of *c*=*c̅*+*c′*. The instantaneous horizontal turbulent flux of odorants was then computed as *u′c′*, as well as the time-averaged mean flux as $\bar{u\prime c\prime }$. Fluxes were computed on the spatial scale of the velocity, after concentration was averaged over the 8 pixel by 8 pixel PIV subwindow. Utilizing 5,000 PIV and 5,000 PLIF images (a total sampling time of ∼200 s) for each flow condition, the temporal average of the horizontal velocity, normalized odorant concentration, and horizontal flux of odorants ( $\bar{u\prime c\prime }$) was computed (Figure 5). The instantaneous measurements of velocity, concentrations, and fluxes encountered by animals navigating in the plume show substantially higher variability than time-averaged values. Since the temporal mean of fluctuations, $\bar{u\prime }$ and $\bar{c\prime }$ is by definition equal to 0, the magnitude of these fluctuations was instead computed as the root mean square (*rms*) of *u* and *c* (Figure 5A,B respectively). This indicates that the magnitude of the horizontal velocity fluctuations were relatively constant throughout the water column, but due to a logarithmic distribution with height of the mean velocity profile, *u′* varied from approximately 45% of *u̅* near the bed (*z* = 0 cm) and decreased to 20% of *u̅* at the elevation of antennules (*z* = 5 cm). *c̅* was highest adjacent to the sand bed and decreased with increased elevation, while the *c′* profile had a parabolic shape. Fluctuations in *c* were approximately 20% of *c̅* near the bed, but increased to approximately 50% of *c̅* at *z* = 5 cm. Turbulent fluxes, $\bar{u\prime c\prime }$, were relatively constant between *z* = 1 cm and 6 cm, but decreased as the bed was approached (*z* < 1 cm) due to enhanced mixing of the plume that decreased the magnitude in *c′*. For *z* > 6 cm, $\bar{u\prime c\prime }$

decreased because the odorant plume had not mixed vertically to this elevation by the time the plume reached 1 m downstream from the source. Since the PIV and PLIF imaging occurred utilizing laser light aligned along a 2D plane, no transverse mixing of the plume (*v′c′*) could be measured.

### 3.2. Numerical Model

**Figure 6.**
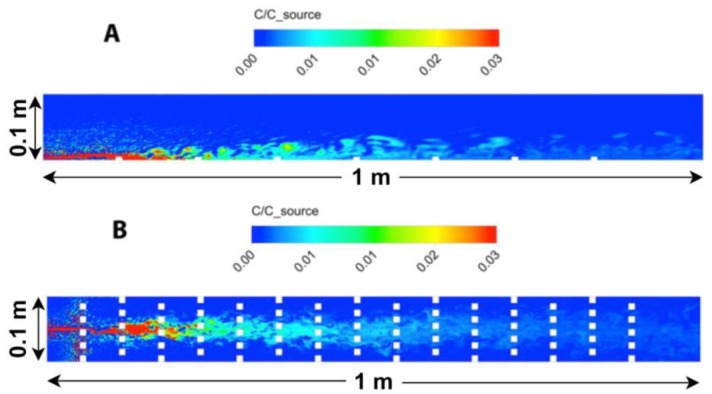
(**A**) Side view of odorant concentration throughout the numerical flume. White squares are roughness elements along the bed; (**B**) Top-down view of odorant concentrations at an elevation of *z* = 0.5 cm. The plume of odorants is released into the flume through a circular point-source inlet located at *z* = 0.3 cm above the bed.

**Figure 7.**
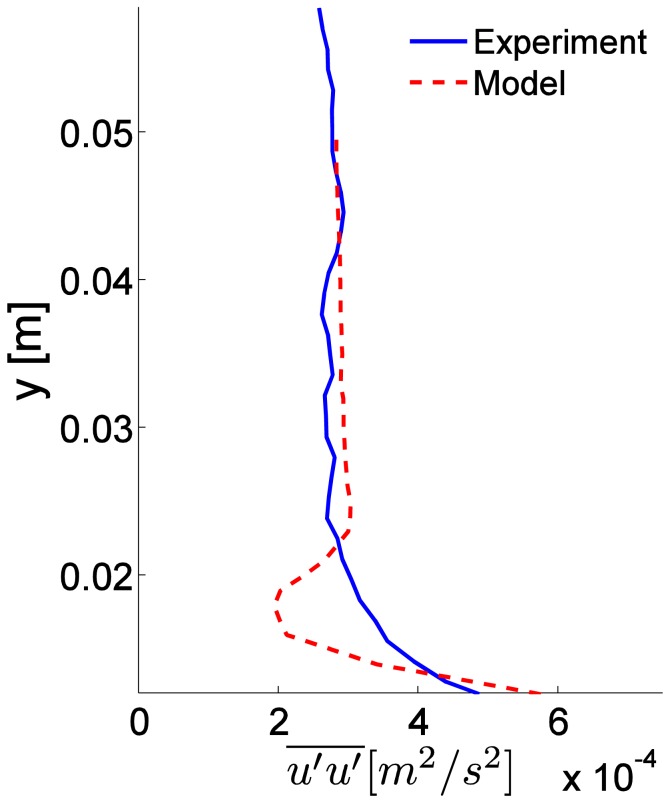
Comparison of horizontal turbulence intensity within the flume experiments and numerical model. Measurements were obtained at 1 m downstream from the source.

A side and top-down view of odorant concentrations obtained from the 3D CFD model is shown in Figure 6. The turbulent stress, $\bar{u\prime u\prime }$, through the water column shows close agreement between experimental and simulated model data (Figure 7A), except for the region adjacent to the bed, where the numerical roughness elements create a notable shear in the flow and a localized increase in $\bar{u\prime u\prime }$. The CFD model aimed to create a turbulent flow structure similar to that measured for the flume studies. Although the sand bed topography cannot be exactly reproduced within a numerical simulation, and thus some deviation of the turbulence structure and thus dispersal characteristics of odors between lab and numerical studies will exist, a benefit of the CFD model is that all three components of velocity, as well as concentration, can be computed at all locations simultaneously within the plume. Smaller odorant concentrations are predicted by the CFD model (∼1% of *c_source_*,Figure 6) compared to the experimental flume data (up to 3% of *c_source_*, Figure 4) because a point source of odorants was used in the CFD simulation, compared to a line source used for the flume experiments that released a greater amount of dye into the flow. Mean flow and turbulence characteristics for the two plumes generated in the CFD model (inlet flow conditions of *U_o_* = 10 and 15 cm s^−1^) are listed in Table 1.

**Figure 8.**
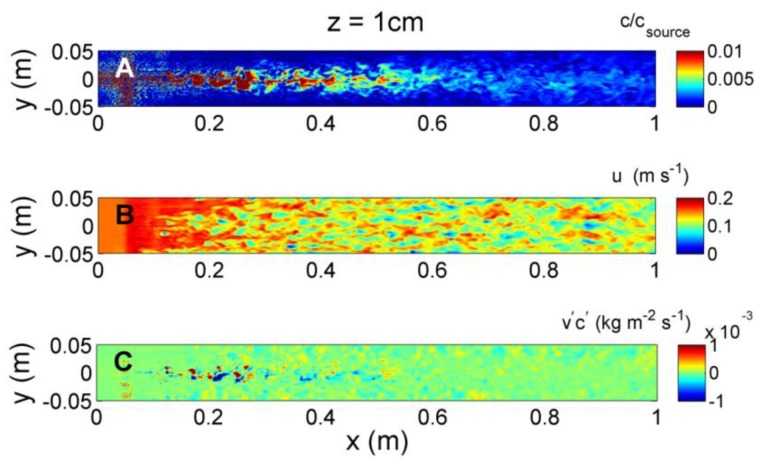
Instantaneous (**A**) odorant concentration; (**B**) horizontal velocity; and (**C**) transverse concentration flux (*v′c′*) at *z* = 1 cm above the flume bed, obtained at *t* = 10 s after initial release of odorant.

Instantaneous odorant concentration, velocity, and transverse flux, *v′c′*, along a horizontal plane *z* = 1 cm above the simulated flume bed are shown in Figure 8. Due to the close proximity of the bed, both the odorant concentrations and velocity structure are significantly impacted by the cube-shaped bedforms, initially causing rapid dispersion of concentration at *x* = 0.05 m due to flow interaction with the first row of bedforms. This dispersion may partially be due to numerical error due to the steep change in roughness when flow first encounters the bedforms. However, this effect is localized and further downstream, the concentration structure evolves slowly, with higher concentrations near the source, and lower, more diffuse concentrations with greater downstream distance. The velocity structure shows uniform conditions along the upstream end of the model flume until the first bedforms occur. Further downstream, distinct turbulence structure is evident as water velocities are directly impacted by the bedforms. The transverse flux shows large variability, both in magnitude along the upstream-downstream, *x*, direction, and in sign (positive or negative) along the transverse, *y*, direction. At *z* = 1 cm, the flux magnitude is greatest near the source, between *x* = 0.1 and 0.4 m downstream, and is on average positive to the left of the plume centerline (*y* > 0 cm), and negative to the right of the plume centerline (*y* < 0 cm), if faced towards the downstream direction.

**Figure 9.**
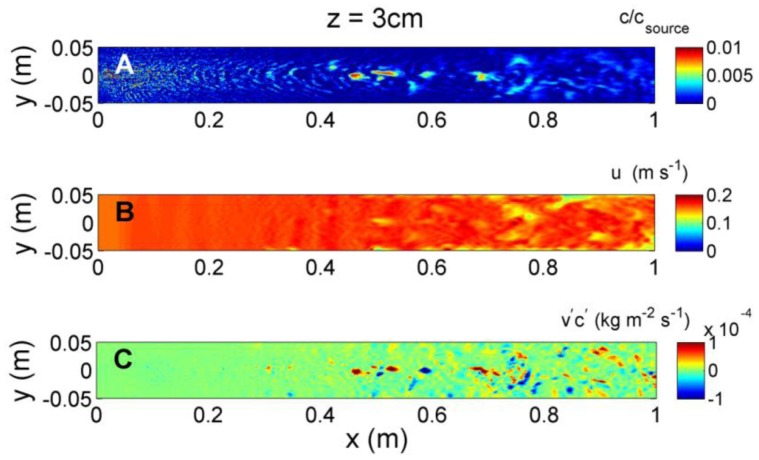
Instantaneous (**A**) odorant concentration; (**B**) horizontal velocity; and (**C**) transverse concentration flux (*v′c′*) at *z* = 3 cm above the flume bed, obtained at *t* = 10 s after initial release of odorant.

**Figure 10.**
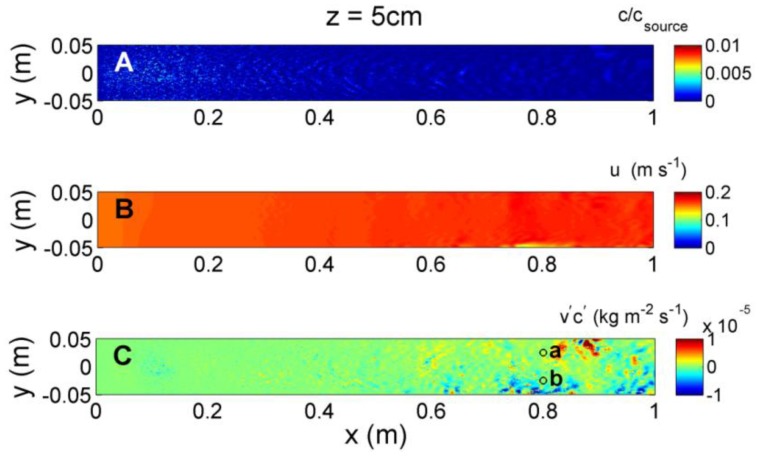
Instantaneous (**A**) odorant concentration, (**B**) horizontal velocity, and (**C**) transverse concentration flux (*v′c′*) at *z* = 5 cm above the flume bed, obtained at *t* = 10 s after initial release of odorant.

At an elevation of *z* = 3 cm (Figure 9), the influence of turbulence generated at the bed is less evident, however, turbulent mixing still injects large fluctuations in both concentration and velocity. The intensity in the fluctuations changes along the upstream-downstream direction, and may provide information to organisms navigating in the plume. However laterally, neither the magnitude in concentration or velocity nor their intermittency give any indication of plume direction laterally across the centerline. Simultaneous measures of odorant and velocity fluctuations do show distinct signals of lateral direction of the plume, with negative fluxes to one side, and positive fluxes to the other side of the plume centerline. At *z* = 5 cm (Figure 10), the transverse flux increases in magnitude at greater downstream distances and spreads laterally compared to *z* = 1 or 3 cm. As is evident from Figures 8, 9 and 10, as the source is approached the location of the largest magnitudes of transverse fluxes occur at a lower elevation and closer to the plume centerline, but remain on average negative fluxes for *y* < 0 and positive fluxes for *y* > 0. This suggests that changes in the magnitude and spread of the transverse flux can offer information about both the transverse and downstream distance from the source that neither the concentration nor ambient velocity can provide independently.

### 3.3. Temporal and Spatial Averaging of the Plume

**Figure 11.**
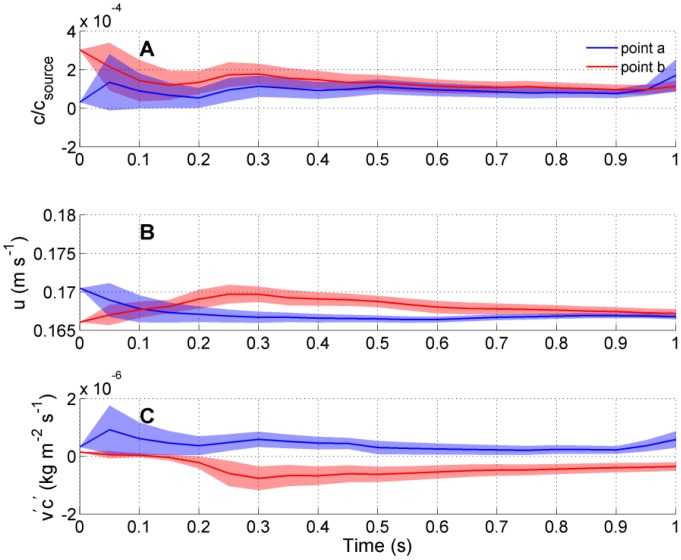
(**A**) Normalized concentration; (**B**) horizontal velocity, u; and (**C**) transverse flux (*v′c′*) at points **a** and **b** (as indicated in Figure 10C) for a 1 s time period. The solid lines show the cumulative mean and the shaded regions show the ±1 standard error around the cumulative mean.

The cumulative mean concentrations, velocities, and transverse fluxes sampled at two points and separated by 5 cm that span the centerline of the plume (at *y* = 2.5 cm and −2.5 cm and a downstream location of *x* = 0.8 m) are shown in Figure 11. This is information that an organism, sampling at an elevation of *z* = 5 cm and utilizing two antennules spaced a distance of 5 cm (which is spacing observed for blue crab antennules [3]), could conceivably quantify if it remained stationary at this location. The cumulative mean converges, and the standard error becomes reduced the longer the organism samples at the same location. This suggests that little information can be statistically gained about the transverse location of the source from differencing values across the plume centerline if just concentration (Figure 11A) is quantified. Although variations in horizontal velocity (Figure 11B) do occur at the two locations due to turbulent eddies, no statistical difference in velocity occurs after averaging for periods longer than ∼0.7 s. However, statistically significant differences in transverse flux can be quantified even after sampling for as little as 200 ms at this location, and these differences remain statistically significant for time periods >1 s. Although the length of time needed to determine statistically different transverse fluxes is dependent upon the location within the plume and initial concentration measured, typically the fluxes show statistical distinct values in <0.5 s, while velocity and concentration tend to statistically converge over a similar length of time.

**Figure 12.**
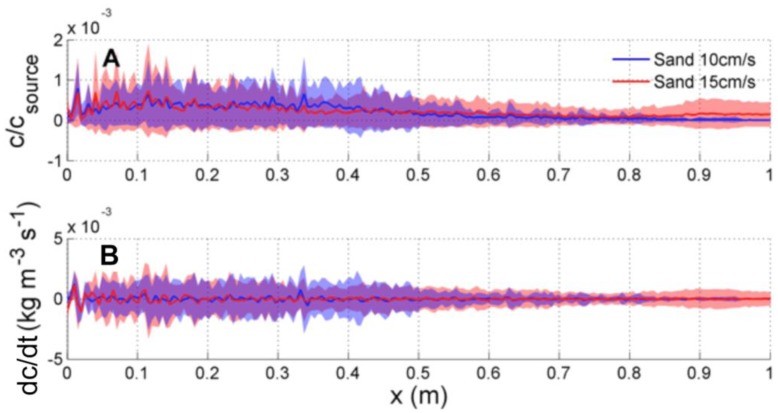
(**A**) Normalized concentration and (**B**) temporal rate of change of concentration (*dc*/*dt*) along the centerline through the length of the flume at *z* = 5 cm. The solid line shows mean values and the shaded region shows the region of ±1 standard deviation around the mean.

The time-averaged concentration field as a function of downstream distance along the plume centerline is shown in Figure 12A for both the *U_o_* = 10 cm s^−1^ and 15 cm s^−1^ mean flow scenarios. Mean values of *c*/*c_source_* (solid red and blue lines) increase slowly as the source is approached, but do not change appreciably due to changes in the mean flow. However, larger fluctuations in concentration occur as the source is approached, indicating that the plume becomes more intermittent. Figure 12B shows the concentration slope, computed as the temporal rate of change of concentration (*dc*/*dt*). For both flow conditions, larger fluctuations in *dc*/*dt* also occur as the source is approached. However, mean and fluctuating estimates in both *c*/*c_source_* and *dc*/*dt* evolve slowly with downstream distance, and either long sampling times, or large movements in upstream-downstream distance are necessary to statistically see changes in plume dynamics that might aid in locating the source.

## 4. Discussion

Results indicate that simultaneous detection of flow and odorants within a turbulent plume can aid aquatic organisms in tracking a source. To develop this link between signal detection, potential tracking behavior and odorant signal properties, we utilized flume experiments that simultaneously quantified spatial variability in both velocity and concentration over a bed composed of sand. We utilized results from the laboratory experiments to develop a 3-dimensional computational fluid dynamics model of a similar turbulent plume, and developed the model for two flow conditions, with inlet velocities *U_0_* =10 cm s^−1^ and 15 cm s^−1^. Our results, as well as results from other studies [8,20,47], suggest that time-averaged concentration as well as the slope, computed as the concentration gradient with respect to time (*dc*/*dt*), converge slowly, and the length of sampling period by an animal too great, to be effectively used in determining position in a plume relative to the source. However, instantaneous fluctuations in concentration, in combination with transverse variations in velocity, vary on sufficiently short timescales to be effectively used to determine directional location of the plume source, potentially by a variety of aquatic organisms [38,48,49]. Specifically, the transverse flux of odorants, *v′c′*, vary both in magnitude and sign, depending on whether the flux is sampled to the left or right of the centerline of the plume. In addition, the transverse difference in magnitude and sign of the flux converge towards the centerline of the plume, and at lower elevations above the bed, as the source is approached. These dynamics suggest that animals, if tracking the edge of the plume where transverse fluxes are largest, can utilize variations in flux magnitude to aid in determining both the lateral location, and downstream distance from the source. Since the sign of the flux also changes depending upon the left or right hand side of the plume relative to the centerline, bilateral comparisons utilizing two antennules are not needed to determine correct orientation within a plume. This agrees with previous findings that the use of only one antennule is sufficient for chemo-orientation within a plume [50], although the use of bilateral comparisons have been shown to enhance the effectiveness and speed of the search [51]. There are many reported search strategies that organisms employ to undergo search, including odor gated rheotaxis where an animal moves upstream as long as sufficient contact with odor is maintained, or eddy chemo-rheotaxis where an animal steers towards a source using eddies containing higher concentrations of odorant (reviewed in [8,48]). However, many organisms, including crabs and some lobster species, appear to track lateral edges of the plume (*i.e*., plume edge tracking [48]) and move towards the centerline as the source is approached [38,49] thus moving along a direction indicating that transverse flux information might be utilized.

Although integration of flow and concentration cues on timescales between 0.1 and 1 s have been shown in sharks, where both sensing along their lateral line [34] and bilateral odor concentration differences between their nares [52] altered orientation behavior to dispersing odorants, no studies to date have directly linked the ability of crustaceans to integrate flow and odorant information to aid in search on these short timescales. Dickman *et al.* [53] utilized measurements of an instantaneous 3D concentration field surrounding actively tracking blue crabs and found a connection between upstream walking speed and bursts of odorant concentration arriving at the antennule chemosensors. The antennule elevation of the crabs generally changed as crabs lowered or raised their thorax as they moved upstream, while transverse crab movements were in apparent response to the transverse distribution of the odorant concentration field. This study also suggested that asymmetry in the odorant concentration distribution at the elevation of the leg chemosensors (at approximately *z* = 1 cm) was responsible for position adjustments in the transverse direction. Although cross-stream motion is aided by chemical signal inputs to receptors on their walking legs, crabs do make rotational movements in response to chemical signals along their antennules, which determine the crab's body angle with respect to the flow [22].

### 4.1. Neural Responses to Odorants and Flows

Reidenbach and Koehl [13] found that the intermittency in encounter rates of odors is greater when an odor plume is sampled by flicking than that for continuous sampling, giving receptor neurons more time to dis-adapt before encountering the next odor pulse. Adaptation of receptor neurons occurs due to prolonged exposure to odorants, and subsequent responses to odor pulses is reduced or absent [54,55]. Flicking therefore enables sensory neurons to remain sensitive to intermittent changes in odor concentration [27,56]. For the clawed lobster *Homarus americanus*, receptor neurons in the antennule respond, after a latency period of at least 50 ms, with a series of action potential spikes. However, after exposure to odorants for ∼300 ms, spike frequency is reduced, and neurons cease firing after 1,500 ms [28,57]. This adaptation resets the sensitivity of the neurons so that they can respond to transient changes in odor concentration relative to background concentrations. This suggests that lobsters need to be exposed to odorants for at least 50 ms for neurons to respond, but for exposure times greater than 1,500 ms, neurons cease responding to ambient background concentrations. Although intermittency in the odorant concentrations is essential for accurate detection of ambient concentrations, adaptation may be beneficial to allow organisms to respond to concentration changes relative to background. While adaptation to odor exposure is relatively rapid, disadaptation in individual *H. americanus* lobster ORNs averaged 14 s [27]. Longer exposure to background concentrations generated more ORN spikes, but did not increase the initial spike frequency. These findings suggest that certain ORNs, at least in *H. americanus*, are sensitive to changes in the ratio of stimulus concentration to background concentration, and allow the receptors to function with equal sensitivity across at least three orders of magnitude in background concentration [56]. This essentially allows these organisms to respond to changes in concentrations relative to background levels [58]. These relative changes in concentration and velocity relative to mean background conditions are also utilized to determine mathematically the magnitude of instantaneous flux events. This may also suggest why many aquatic organisms are found to track the edges of the plume, where both the transverse flux is largest, and intermittency is increased [13]. In addition, statistical differences in the magnitude of the transverse flux can be obtained on timescales of ≤1 s, which is within the range of neural response times of aquatic animals.

## 5. Conclusions

This study shows that simultaneous sampling of both flow and odorant concentration can provide valuable cues for tracking of odorant plumes compared to sampling of odorants alone. Mean odorant concentration increases as the animal moves towards the plume source, helping it determine the upstream direction. Variability in the temporal rate of change of concentration also increases as the animal moves towards the source. However, both these statistics converge slowly, and therefore may not be useful for aquatic organisms that often make rapid movements while navigating in a plume [8]. Comparison of flux of odorants in the transverse direction between two points on either side of the plume centerline separates statistically on timescales ≤0.5 s, while velocity and concentration tend to converge over similar lengths of time. This difference in flux becomes larger, and converges towards the plume centerline as the animal moves closer to the plume source. Although it is still unknown if crustaceans can integrate variations in odorant concentrations and velocity relative to background levels, our results suggest that flux can be a significant factor enabling animals to orient themselves favorably in the search for a plume source, and occurs on timescales relevant to responses by olfactory neurons. In addition, our results show that transverse flux is a useful parameter to be quantified and incorporated into the design and utilization of artificial bio-mimetic sensors.

## Figures and Tables

**Table 1.** Mean statistics for the simulated flow at inlet flow speeds of U_0_ = 10 and 15 cm s^−1^ respectively. U_o_ is the inlet velocity along the upstream end of the flow domain. *u̅* is the mean velocity over the bed roughness 1 m downstream from the source, u_*_ is the friction velocity, Re_*_ is the roughness Reynolds number computed as Re_*_ = u_*_h/*υ* (h = height of roughness element), η is the Kolmogorov lengthscale, ε is the dissipation rate of turbulence and *λ_B_* is the Batchelor scale.

	*u*** (cm s^−1^)**	u_*_ (cm s^−1^)	Re_*_	***η* (cm)**	***ε* (cm^2^s^−3^)**	***λ****_B_*** (cm)**
Sand, *U_0_* =10 cm s^−1^	10.6	0.15	8.4	0.13	2.5 × 10^−3^	2.9 × 10^−3^
Sand, *U_0_* =15 cm s^−1^	15.2	0.18	10.1	0.10	7.1 × 10^−3^	2.2 × 10^−3^
